# Quercetin regulates autophagy and attenuates airway inflammation in a murine model of asthma: association with PI3K/Akt/mTOR signaling pathway modulation

**DOI:** 10.3389/fphar.2026.1755094

**Published:** 2026-01-30

**Authors:** Liye Lang, Sheng Liu, Weishuai Zhang, Jialin Zhang, Hua Liu

**Affiliations:** Department of Respiratory and Critical Care Medicine, Affiliated Hospital of Nantong University, Medical School of Nantong University, Nantong, China

**Keywords:** asthma, autophagy, inflammation, PI3K signalings, quercetin

## Abstract

**Background:**

As a natural flavonoid, quercetin has anti-inflammatory and anti-oxidative activities. Studies confirm its beneficial effect on asthma, but the underlying mechanism remains unclear. This study aimed to systematically evaluate quercetin’s efficacy in treating asthma, explore its regulatory role in asthma-related autophagy and associated signaling pathways, and provide new insights for asthma treatment research.

**Methods:**

*In vivo*, ovalbumin (OVA)-induced asthma model mice were first successfully established, then randomly assigned to five groups: control, asthma model, low/high-dose quercetin, and dexamethasone positive control. ELISA, histopathological staining, immunohistochemistry and Western blot were used to assess quercetin’s therapeutic effect and molecular mechanism. To complement the *in vivo* findings from the OVA-induced asthmatic mouse model, *in vitro* experiments were conducted using the human bronchial epithelial cell line BEAS-2B. Specifically, the cell line was stimulated with TNF-α and IL-4 to establish an inflammatory model, further validating quercetin’s regulation of autophagy and inflammation.

**Results:**

*In vivo*, quercetin reduced inflammatory cell count and proinflammatory cytokine levels in asthmatic mice’s bronchoalveolar lavage fluid (BALF), lowered serum IgE, and alleviated lung inflammatory infiltration and pathological damage. It also inhibited lung autophagy and activated the PI3K/Akt/mTOR signaling pathway. *In vitro*, consistent with *in vivo* findings, quercetin downregulated proinflammatory factors and autophagy-related proteins in TNF-α/IL-4-stimulated BEAS-2B cells. In addition, the PI3K/Akt/mTOR signaling pathway is also activated by quercetin.

**Conclusion:**

Quercetin attenuates airway inflammation and lung damage in asthmatic mice. Its therapeutic effect is associated with the modulation of PI3K/Akt/mTOR signaling pathway activity and the regulation of excessive autophagy, which provides new potential approaches and mechanistic insights for asthma treatment.

## 1 Introduction

Asthma is a chronic airway inflammatory disease characterized by bronchial hyperresponsiveness and reversible airflow obstruction. Its pathophysiology includes mucosal edema, mucus hypersecretion and bronchial contraction, leading to recurrent wheezing, dyspnea and cough, with characteristic diurnal or seasonal changes (Porsbjerg et al., 2023). The disease has become a global health challenge, and its incidence continues to rise. Epidemiological studies have shown that the total number of asthma patients has exceeded 350 million, and the number of deaths caused by asthma is close to 500,000 cases per year (Kuruvilla et al., 2019, Reynolds et al., 2021). Asthma not only affects the daily life and work of patients, but also brings a huge social and economic burden (Loftus and Wise, 2015). Asthma is highly heterogeneous in clinical manifestations and pathological mechanisms. According to different pathogenesis characteristics, asthma can be divided into multiple phenotypes, and its internal molecular mechanisms are also significantly different. Among them, immunoglobulin E (IgE)-mediated allergic asthma is the most common clinical subtype (Wenzel, 2012, Akar-Ghibril et al., 2020) and its pathogenesis involves the activation of T help 2 (Th2) cells, IgE-mediated mast cell degranulation and eosinophil infiltration, eventually leading to persistent cell infiltration (Komlosi et al., 2022).

Autophagy is a highly conserved cellular metabolic process that selectively degrade intracellular substances by forming a specific bilayer membrane structure and autolysosome to achieve recycling and homeostasis of cell components (Yu et al., 2018). Under normal physiological conditions, autophagy is beneficial to cell survival. However, increased autophagic activity induced by pathological stimuli such as oxidative damage and inflammatory mediators can disrupt cellular homeostasis and promote the development of many diseases (Levine and Kroemer, 2008, Liu et al., 2023). Studies have shown that excessive autophagy leads to immune response disorders and aggravates inflammatory diseases (such as asthma) (Theofani and Xanthou, 2021, Dong et al., 2023). In asthmatic mouse models specifically, high expression of autophagy-related proteins was detected in lung tissues (Liu et al., 2016). Compared with normal or non-severe individuals, the levels of autophagy markers in peripheral blood cells, peripheral blood eosinophils, and bronchial fibroblasts were increased in patients with severe asthma (Ban et al., 2016, Ramakrishnan et al., 2020). It is worth noting that targeting mTOR-mediated autophagy inhibition effectively reduced asthma-related pathology and significantly reduced airway inflammation and mucus hypersecretion in the experimental model (Yang et al., 2015). These findings highlight autophagy regulation as a promising strategy for asthma treatment.

The PI3K/Akt/mTOR pathway plays an important role in the regulation of autophagy, and its activation can inhibit the function of autophagy initiation complex ULK1 through mTOR-dependent pathway (Kim and Guan, 2015). Specifically, when the PI3K/Akt signaling pathway is activated, Akt can directly phosphorylate mTOR at Ser 2,448 and enhance the activity of mTORC1, thereby inhibiting the ULK1 initiation complex to block autophagy (Islam et al., 2021, Glaviano et al., 2023). In addition, this pathway plays an important regulatory role in inflammation and is often used as a potential therapeutic target for various diseases (Yu and Cui, 2016). Experimental studies have found that the phenotype of allergic asthma, including increased mucus production and airway hyperresponsiveness aggravated when the PI3K/Akt/mTOR signaling pathway was inhibited (Zou et al., 2018). In contrast, activation of it can effectively inhibit autophagy in lung tissue, thereby reducing airway inflammation (Wang et al., 2021).

Glucocorticoid is a clinical first-line treatment for allergic asthma, but its long-term use can easily lead to drug resistance and adverse reactions, and this therapeutic dilemma necessitates the exploration of alternative treatment strategies with improved safety profiles. Quercetin is a natural flavonoid compound, whose unique chemical structure makes it have significant anti-inflammatory, antioxidant and immunomodulatory activities (Carrillo-Martinez et al., 2024, Li et al., 2016), and has been initially confirmed to have a therapeutic effect on asthma. Studies have shown that quercetin can inhibit airway hyperresponsiveness in asthma models, reduce inflammatory cell infiltration, and down-regulate the secretion of cytokines (Park et al., 2009, Rogerio et al., 2007). However, the specific mechanism of its treatment of asthma has not been fully elucidated. Existing research has indicated that quercetin can regulate autophagy and PI3K/Akt signaling pathway (Li et al., 2023, Yu et al., 2024). But there is no clear evidence to show whether quercetin alleviates asthma airway inflammation by interfering with autophagy, especially whether it involves the regulatory mechanism of PI3K/Akt/mTOR. Therefore, the purpose of this study was to explore the effect of quercetin on airway inflammation in asthmatic mice, and to analyze its effect on autophagy activity in lung tissue and its regulation on PI3K/Akt/mTOR pathway, in order to provide a new direction for natural drug intervention in asthma.

## 2 Materials and methods

### 2.1 Animals and treatment

**FIGURE 1:**
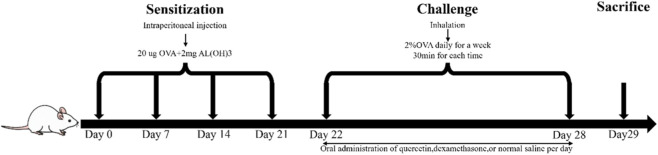
Modeling flow chart of asthmatic mice.

This study was conducted in accordance with protocols approved by the Institutional Animal Care and Use Committee of Nantong University (Authorization Number: S20241110-001). Forty balb/c mice (6-week-old, female) were randomly assigned to five experimental groups by simple randomization: negative control (Con, 0.9% NaCl), ovalbumin (OVA)-induced asthmatic model (Ast), low-dose quercetin treatment (Qct-L, 10 mg/kg), high-dose quercetin treatment (Qct-H, 20 mg/kg), and Positive control (Dex, 1 mg/kg dexamethasone). The selection of quercetin doses (10 mg/kg and 20 mg/kg) was determined by referencing to previous relevant studies (Wang et al., 2021, Park et al., 2009, Fang et al., 2023) and integrating the preliminary experimental results of this study. Next, we established an OVA-induced allergic asthma model using OVA (Grade V, Sigma-Aldrich) combined with aluminum hydroxide adjuvant (InvivoGen). The experimental protocol consisted of two phases (Figure 1). During the sensitization phase on days 0, 7, 14 and 21, mice received intraperitoneal injections containing 20 μg of ovalbumin and 2 mg of aluminum hydroxide. In the subsequent challenge phase from day 22 to day 28, animals were exposed daily to 2%OVA via aerosol inhalation for 30 min. Throughout the challenge period, mice in treatment groups received 10 mg/kg quercetin (purity≧98%, Shanghai Yuanye Biotechnology), 20 mg/kg quercetin or 1 mg/kg dexamethasone (purity≧99%, Beyotime Biotechnology), and mice in Con and Ast groups were given equivalent volumes of normal saline. All compounds were administered by oral gavage except for the intraperitoneal sensitization and aerosolized ovalbumin challenges.

### 2.2 Inflammatory cell count and cytokine detection

On day 29, mice were anesthetized using tribromoethanol (1.25%, Nanjing Aibei Biotech). Bronchoalveolar lavage was performed with 300 μL precooled PBS, and this procedure repeated three times. BALF was centrifuged (1000 × g, 10 min, 4 °C) while blood samples were collected and centrifuged (3,000 rpm, 30 min, 4 °C) after 1 h standing. Supernatants were stored at −80 °C for cytokine analysis. The levels of IL-4, IL-13, IFN-γ and OVA-specific IgE were quantified using ELISA kits (Jingmei Biotechnology, Jiangsu, China). Cells were classified and counted with Wright-Giemsa staining (Solarbio, Beijing, China). The cells were classified and counted under a microscope, with particular attention to the infiltration of eosinophils, neutrophils and lymphocytes.

### 2.3 Pathological observation of lung tissue

After the completion of bronchoalveolar lavage, lung tissue samples were taken immediately and fixed with 4% paraformaldehyde for 24 h. Subsequently, the samples were subjected to gradient ethanol dehydration, xylene transparency treatment, and finally paraffin embedding to prepare tissue sections with a thickness of 4 μm. Hematoxylin and Eosin (H&E) staining was used to observe lung tissue, and digital micrographs of three randomly selected fields were acquired for each specimen. The intensity of inflammatory cell infiltration in pulmonary tissues was evaluated by double-blind method and standardized grading scale (Zhou et al., 2023), with the mean of triplicate measurements constituting the sample’s inflammatory score.

### 2.4 Immunohistochemistry

Lung tissue samples were dewaxed until hydration, and antigen repair was performed using 0.01M sodium citrate buffer. Tissue sections were incubated with the primary antibody overnight. The primary antibody LC3B (1:200, Proteintech, Wuhan, China) was incubated overnight in a wet box at 4 °C, and the secondary antibody was incubated with HRP-conjugated goat anti-rabbit IgG (1:200 dilution, 1 h, RT). Following, tissue sections were processed through DAB chromogenic development and hematoxylin counterstaining. After three thorough washes in phosphate-buffered saline, specimens were permanently mounted using resin-based medium. The area of interest (AOI) was defined under a microscope, followed by measurement of integrated optical density (IOD) using Image-Pro Plus image analysis software. These values were computationally normalized to generate average optical density (AOD = IOD/AOI) for comparative quantification.

### 2.5 Western blotting

RIPA buffer (Solarbio, Beijing, China) supplemented with protease phosphatase inhibitors was used to extract proteins from lung tissue and cultured cells. The protein samples were separated by sodium dodecyl sulfate-polyacrylamide gel electrophoresis (SDS-PAGE), and transferred to a 0.45 μm pore size polyvinylidene fluoride (PVDF) membrane using a semi-dry transfer system (Trans-Blot SD, Bio-Rad). Next, the PVDF membrane was blocked with TBS-Tween20 buffer (TBST) containing 5% skimmed milk powder on a shaker (RT, 60 rpm) for 60 min. Subsequently, the membrane was placed in primary antibodies and incubated overnight at 4 °C. After been incubating with HRP-conjugated secondary antibodies (1:1000, 1 h, RT), chemiluminescent signals were captured and quantified using ImageJ. The antibodies used in the experiment included: LC3B (1:1000, Proteintech,Wuhan, China), Beclin-1 (1:1000, Proteintech, Wuhan, China), PI3K (1:1000, Affinity Biosciences, Changzhou, China), p-PI3K(1:1000, Affinity Biosciences, Changzhou, China), Akt (1:1000, Affinity Biosciences, Changzhou, China), p-Akt (1:1000, Affinity Biosciences, Changzhou, China), mTOR (1:1000, Affinity Biosciences, Changzhou, China), p-mTOR (1:1000, Affinity Biosciences, Changzhou, China).

### 2.6 Cell viability and treatment

The human bronchial epithelial cells BEAS-2B (Beyotime Biotechnology, Shanghai, China) used in this experiment were cultured in DMEM/F12 complete medium (containing 10% FBS + 1% double antibody). To evaluate quercetin’s cytotoxicity, BEAS-2B cells in logarithmic growth phase were trypsinized and seeded at 5 × 10^3^ cells/well in 96-well plates. Cells were maintained in a humidified 5% CO_2_ incubator at 37 °C for 6–8 h to ensure proper adherence. Six quercetin concentrations (0–80 μM) were tested alongside blank (medium only) and negative (cells and medium) controls, with triplicate wells per condition. After 24-h treatment, 10 μL CCK-8 reagent (Abbkine, Wuhan, China) was added per well followed by 2–4 h incubation. Absorbance at 450 nm was measured, with viability calculated as (OD _treatment_–OD _blank_)/(OD _negative control_–OD _blank_) × 100%. The safety threshold was defined as ≥90% viability by nonlinear regression analysis. Upon reaching logarithmic growth, cells were trypsinized and seeded in 6-well plates (1 × 10^5^ cells/well). Based on CCK-8 results, eight groups were established: Control, 0.1% DMSO, TNF-α/IL-4 (10 ng/mL each), quercetin (10/20 μM), quercetin (10/20 μM) + LY294002, and dexamethasone (1 μM). After cell adherence, pretreatment was performed: quercetin/dex for 1–2 h, DMSO solvent control, or no treatment (Control). All groups except Control were stimulated with TNF-α/IL-4 (Novoprasen, China) for 24 h to induce inflammation, and LY294002 was used to inhibit the PI3K/Akt/mTOR signaling pathway in the inhibitor groups. ELISA was used to detect inflammatory factors such as IL-5 and IL-6 (Jingmei Biotechnology, Jiangsu, China) and Western blot was used to analyze autophagy marker proteins (LC3, Beclin-1) and PI3K/Akt/mTOR signaling pathway.

### 2.7 Statistical analysis

Data analysis was conducted with GraphPad Prism 9.0. Normality and variance homogeneity were confirmed with the Shapiro-Wilk and Levene’s tests, respectively. One-way ANOVA followed by Tukey’s multiple comparisons test was used to assess differences among groups. Results are presented as mean ± SD, and *P* < 0.05 denoted significance.

## 3 Results

### 3.1 Effects of quercetin on inflammatory cells, cytokines of BALF and serum IgE in asthmatic mice

**FIGURE 2:**
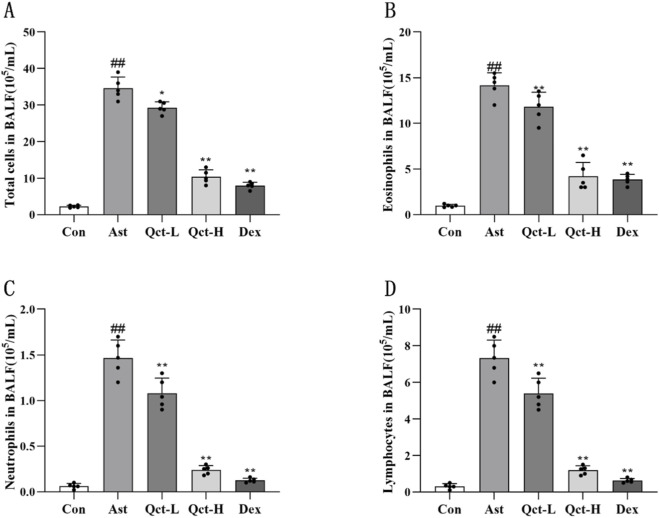
Quercetin modulates inflammatory cell profiles in the BALF of asthmatic mice. Differential cell counts for **(A)** total leukocytes, **(B)** lymphocytes, **(C)** eosinophils, and **(D)** neutrophils *N* = 5/group. #*P* < 0.05, ##*P* < 0.01 versus Con group; *P < 0.05, **P < 0.01 versus Ast group.

**FIGURE 3:**
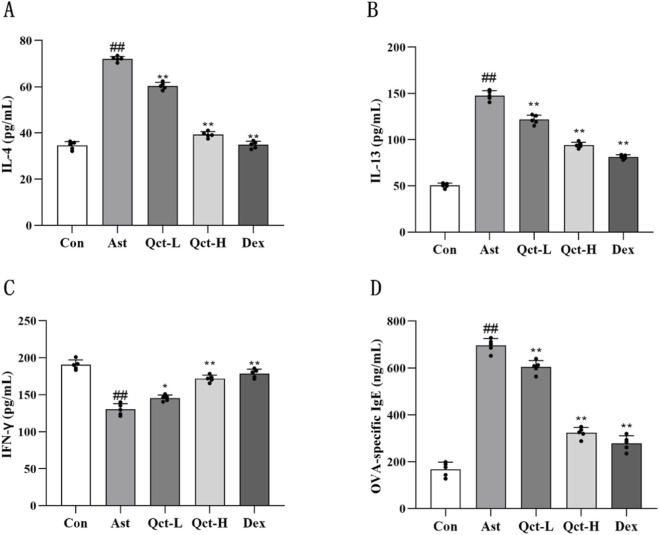
**(A–C)** The regulatory effect of quercetin on cytokine levels in BALF of asthmatic mice. **(D)** Effects of quercetin on serum OVA -specific IgE levels in asthmatic mice. *N* = 5/group. #*P* < 0.05, ##*P* < 0.01 vs. Con group; *P < 0.05, **P < 0.01 vs. Ast group.

Figure 2 shows that, compared with the Con group, the total number of inflammatory cells, eosinophils, neutrophils and lymphocytes in the Ast group increased significantly (*P* < 0.01). On the contrary, the treatment of quercetin and dexamethasone reduced these changes (*P* < 0.05), and this trend is more significant in the Qct-H group and the Dex group. Next, we used ELISA to detect the supernatant of BALF. It can be seen from Figures 3A–C, in the Ast group, the levels of IL-4 and IL-13 were significantly increased compared to the Con group (*P* < 0.01), while the trend of IFN-γ was opposite (*P* < 0.01), which is consistent with the typical characteristics of Th2 immune disorders in asthma. Through the therapy of quercetin and dexamethasone, the levels of IL-4 and IL-13 significantly decreased (*P* < 0.05), with the level of IFN-γ increasing at the same time (*P* < 0.05). Next, we detected serum IgE levels by ELISA and found that (Figure 3D), the level of serum OVA-specific IgE in the Ast group significantly increased compared to the Con group (*P* < 0.01). However, the levels of serum OVA-specific IgE in each treatment intervention group were significantly reduced compared to the Ast group (*P* < 0.05). Among them, the decrease trend of serum OVA-specific IgE level in Qct-H group and Dex group was more significant.

### 3.2 Quercetin alleviated the inflammatory infiltration and pathological damage of lung tissue in asthmatic mice

**FIGURE 4:**
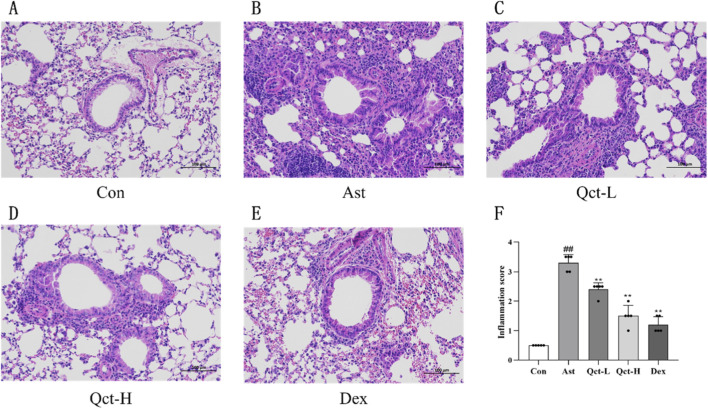
Histopathological evaluation of pulmonary tissue architecture across experimental groups. **(A–E)** Histopathological observation of H&E staining. Scale bar: 100 μm (20 × ). **(F)** Lung tissue inflammation score. *N* = 5/group. #*P* < 0.05, ##*P* < 0.01 versus Con group; *P < 0.05, **P < 0.01 versus Ast group.

H&E staining was performed to evaluate quercetin’s impact on lung inflammation and structural alterations in asthmatic mice. It can be seen from Figures 4A–F that the lung tissue structure of the Con group was complete, the bronchial epithelial cells were arranged regularly, the alveolar septum was uniform, and no obvious inflammatory infiltration was observed. In contrast, the lung tissue of the Ast group showed significant thickening of the bronchial wall and obvious stenosis of the bronchial lumen, with a large number of inflammatory cells infiltrating around it (*P* < 0.01), accompanied by pathological changes such as widened alveolar septum and partial alveolar collapse. After treatment with the high-dose quercetin and dexamethasone, the above pathological changes were significantly alleviated. The results of semi-quantitative analysis showed that compared with the Ast group, the inflammation scores of the Qct-H group and the Dex group were significantly reduced (*P* < 0.01). It is noteworthy that the inflammatory performance of the Qct-L group was also improved to a certain extent compared with the Ast group, and the difference identified by semi-quantitative analysis was statistically significant (*P* < 0.01).

### 3.3 Quercetin inhibited lung tissue’s autophagy in asthmatic mice

**FIGURE 5:**
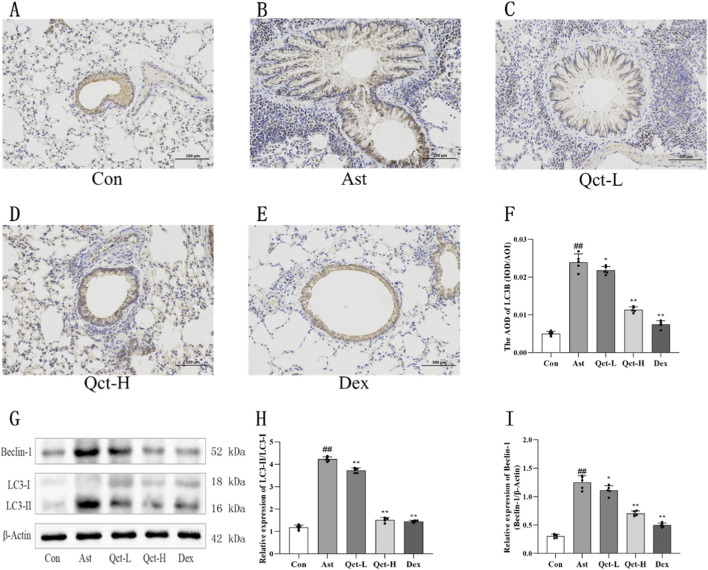
Quercetin modulates autophagy-related protein expression in murine asthma model. **(A–E)** Immunohistochemistry of LC3B in each group. Scale bar: 100 μm (20 × ). **(F)** Immunohistochemical quantification. *N* = 5/group. **(G–I)** Western blot analysis of LC3B and Beclin-1 protein levels. *N* = 5/group. #*P* < 0.05, ##*P* < 0.01 versus Con group; *P < 0.05, **P < 0.01 versus Ast group.

LC3B is a key indicator to evaluate the level of autophagy (Cavalli and Cenci, 2020). In order to comprehensively and systematically explore quercetin’s effect on lung tissue autophagy in asthmatic mice, this study used immunohistochemical staining and Western blotting to analyze LC3B expression (Figure 5). According to the experimental results, LC3B protein was weakly positively stained in the lung tissue of the Con group, which means that its basic autophagy level is in physiological homeostasis (Figures 5A–E). In contrast, LC3B staining intensity was notably enhanced in lung tissue samples from the Ast group, which indicated that the autophagy activity of lung tissue was abnormally increased in the pathological state of asthma. After quercetin intervention, the expression of LC3B in lung tissue of mice in the experimental group showed a downward trend. Immunohistochemical quantification (Figure 5F) revealed that the AOD of LC3B in the Qct-H group and Dex group significantly decreased compared to the Ast group (*P* < 0.01), and the expression levels of LC3B in these two groups were close to the Con group.

The results of Western blotting further verified the above findings (Figures 5G–I). These results demonstrated a significantly elevated LC3-II/LC3-I ratio (*P* < 0.01) and increased Beclin-1 protein levels (*P* < 0.01) in the lung tissues of the Ast group compared to the Con group. After quercetin intervention, the LC3-II/LC3-I ratio and Beclin-1 expression were reduced (*P* < 0.05), especially in the Qct-H group and Dex group.

### 3.4 Quercetin activates PI3K/Akt/mTOR signaling pathway in the lung tissue of asthmatic mice

**FIGURE 6:**
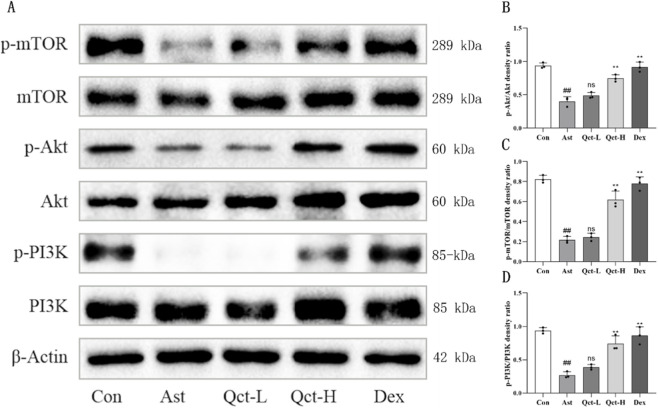
Quercetin can activate PI3K/Akt/mTOR signaling pathway in lung tissue of asthmatic mice. **(A)** Western blot results of PI3K, p-PI3K, Akt, p-Akt, mTOR and p-mTOR proteins; **(B–D)** Quantitative analysis of the p-PI3K/PI3K, p-Akt/Akt and p-mTOR/mTOR protein ratios. N = 3/group. #*P* < 0.05, ##*P* < 0.01 versus Con group; #*P* < 0.05, **P < 0.01 versus Ast group. ns indicates no statistically significant difference compared with the Ast group.

Previous studies point out that the activation of the PI3K/Akt/mTOR pathway can directly inhibit the occurrence of autophagy (Ba et al., 2019). Based on this, we detected the expression of the signaling pathway-related proteins in the lung tissues of mice. The results of Western blot (Figure 6) showed that the levels of phosphorylated PI3K (p-PI3K), phosphorylated Akt (p-Akt) and phosphorylated mTOR (p-mTOR) in the lung tissue of the Ast group were significantly lower than those in the Con group. The levels of p-PI3K, p-Akt and p-mTOR in the Qct-H group and Dex group were significantly increased compared to the Ast group (P < 0.05). However, the increase in p-PI3K, p-Akt, and p-mTOR levels observed in the Qct-L group did not reach statistical significance.

### 3.5 Quercetin reduces the inflammatory levels in TNF-α/IL-4 co-stimulated BEAS-2B cells

**FIGURE 7:**
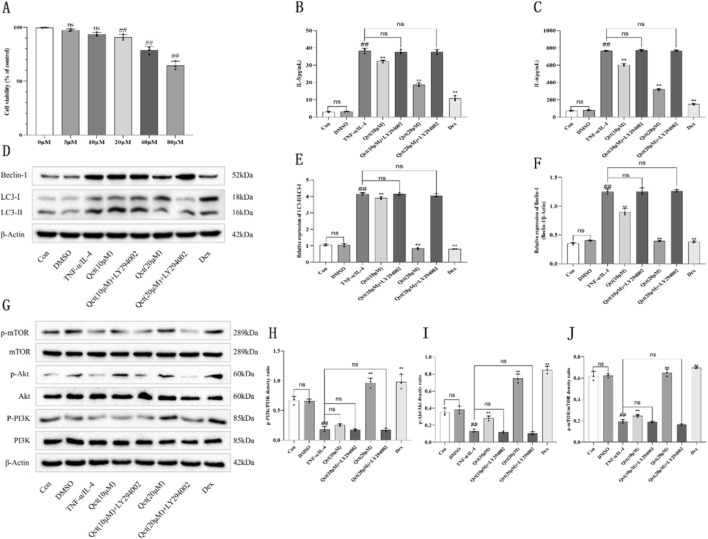
Effects of quercetin on the levels of inflammation, autophagy and the PI3K/Akt/mTOR signaling pathway in BEAS-2B cells stimulated by TNF-α/IL-4. **(A)** Cell viability. **(B,C)** Quercetin reduced the level of inflammation in BEAS-2B cells stimulated by TNF-α/IL-4. **(D–F)** Quercetin reduced the expression of autophagy protein in BEAS-2B cells stimulated by TNF-α/IL-4. **(G–J)** Quercetin activates PI3K/Akt/mTOR signaling pathway in BEAS-2B cells stimulated by TNF-α/IL-4. N = 3/group. #*P* < 0.05, ##*P* < 0.01 versus Control group; *P < 0.05, **P < 0.01 versus TNF-α/IL-4 group. ns indicates no statistically significant difference.

In addition, we used BEAS-2B cells for *in vitro* experiments to further verify the results of *in vivo* experiments. Before this, the CCK-8 experiment was used to establish the safe drug concentration of quercetin (Figure 7A). When the drug concentration exceeded 20 μM, cell viability showed a statistically significant decline but remained at approximately 90%. When the drug concentration reached 40 μM, cell viability decreased sharply, indicating that quercetin at this concentration exerts severe cytotoxicity on the cells. Therefore, we selected two concentrations (10 μM and 20 μM) for subsequent experiments. By detecting the levels of inflammatory factors in the supernatant of BEAS-2B cells, we found that there was no statistical difference in the expression levels of inflammatory factors in the DMSO group compared with the control group, which excluded the additional effects of the use of solvents on cell experiments. The levels of IL-5 and IL-6 in the TNF-α/IL-4 group were significantly higher than those in the control group. The expression levels of inflammatory factors in the treatment group treated with 10 μM quercetin were slightly lower than those in the TNF-α/IL-4 group, and the difference was statistically significant. However, treatment with 20 μM quercetin and dexamethasone significantly reduced the expression levels of these cytokines. It is worth noting that the use of LY294002 significantly reversed the therapeutic effect of quercetin (Figures 7B,C).

### 3.6 Quercetin modulates autophagy in association with PI3K/Akt/mTOR signaling pathway in TNF-α/IL-4 co-stimulated BEAS-2B cells

In order to further explore the potential association and regulatory effect of quercetin on autophagy and PI3K/Akt/mTOR signaling pathway, this study used quercetin to treat BEAS-2B cells stimulated by TNF-α/IL-4 combined induction, and detected the level of autophagy protein. In addition, LY294002 was added to reverse the activation of PI3K/Akt/mTOR signaling pathway. The results showed that the ratio of LC3-II/LC3-I and the level of Beclin-1 in the TNF-α/IL-4 group were significantly increased compared to the blank control group (*P* < 0.01), suggesting that inflammatory stimulation could significantly enhance the autophagy activity of cells. After treatment with 20 μM quercetin, the level of autophagy significantly decreased (*P* < 0.01). This result was highly consistent with the results of animal experiments, which further confirmed that quercetin can exert anti-inflammatory effects by inhibiting autophagy (Figures 7D–F). However, the use of LY294002 significantly reversed the inhibitory effect of quercetin on autophagy in BEAS-2B cells. Figures 7G–J further show that the PI3K/Akt/mTOR signaling pathway in the asthma group was significantly inhibited compared with the control group (*P* < 0.01). After treatment with quercetin, the pathway was significantly activated (*P* < 0.01). LY294002 significantly reversed the activation of PI3K/Akt/mTOR signaling pathway by quercetin (*P* < 0.01). These results indicate that quercetin can modulate autophagy in association with altered activity of the PI3K/Akt/mTOR signaling pathway.

## 4 Discussion

This study confirmed the therapeutic effect of quercetin on asthma and its regulatory mechanism of autophagy in asthma. In animal experiments, the asthmatic group demonstrated characteristic type 2 immune polarization, evidenced by elevated Th2-associated cytokine production, increased BALF leukocyte infiltration, and OVA-specific IgE titers, alongside suppressed IFN-γ expression, indicating that the asthma model was successfully prepared.

Notably, our findings are consensus with previous studies while providing further extensions. Zhang et al. reported that quercetin alleviates airway remodeling by blocking the TGF-β1/Smad-periostin axis (Fang et al., 2023), whereas Li et al. emphasized its role in mitigating neutrophilic inflammation through inhibiting ferroptosis and M1 macrophage polarization (Wang et al., 2023). Different from these studies focusing on structural remodeling or inflammatory cell metabolism, our research primarily focuses on the potential association between the PI3K/Akt/mTOR signaling pathway, autophagy, and the anti-asthmatic effect of quercetin. Consistent with previous reports (Hussein et al., 2023, Du et al., 2016, Wu et al., 2017), we found that autophagy levels were increased in the lung tissues of OVA-induced asthmatic mice (evidenced by upregulated LC3B); more importantly, we further observed that high-dose quercetin could significantly reverse this overactivated autophagy (characterized by decreased LC3-II/LC3-I ratio and Beclin-1 expression) while concurrently attenuating airway inflammation. Further, the therapeutic effects of quercetin in this study exhibited a distinct dose-dependent profile. Low-dose quercetin exerted statistically significant yet moderate effects, whereas high-dose quercetin induced more robust modulation of the PI3K/Akt/mTOR-autophagy axis and anti-inflammatory activity. This characteristic is consistent with previous reports on flavonoid compounds and highlights the importance of dose optimization for maximizing the therapeutic potential of quercetin in asthma treatment. This result was also validated in BEAS-2B cells stimulated with TNF-α/IL-4. These observations suggest that the anti-asthmatic effect of quercetin is partially achieved by inhibiting overactivated autophagy, thereby complementing the previously reported TGF-β1/Smad and ferroptosis-related mechanisms.

It is generally believed that Th2 cytokines play a dominant role in allergic asthma, a phenomenon manifested by excessive activation of Th2 cytokines (such as IL-4, IL-13) and inhibition of Th1-type responses (such as IFN-γ) (Leon and Ballesteros-Tato, 2021, Caminati et al., 2018, Olsthoorn et al., 2025). In this study, OVA-induced asthmatic mice showed typical Th2 immune inflammation characteristics. The levels of IL-4 and IL-13 in BALF significantly increased, while the level of IFN-γ decreased, which was consistent with the above characteristics. This study showed that quercetin significantly reduced airway inflammation in asthmatic mice by down-regulating the levels of IL-5, IL-13 in BALF and OVA-specific IgE in serum and up-regulating the level of IFN-γ. *In vitro*, the intervention of quercetin reduced the levels of IL-5 and IL-6 factors in the supernatant of BEAS-2B cells, which further supports its therapeutic effect *in vivo*.

Previous studies have confirmed that OVA challenge potently triggers autophagy activation in lung tissues of mice (Peng et al., 2024). In addition, compared with normal people, the levels of autophagy markers in sputum granulocytes, peripheral blood cells, peripheral blood eosinophils and bronchial fibroblasts in patients with severe asthma were significantly increased (Islam et al., 2021, Glaviano et al., 2023). This finding provides a new perspective for further understanding the pathogenesis of asthma. Based on this, many studies have further revealed the role of Th2 cytokine IL-13 in the process of autophagy. Studies have found that IL-13 can activate the autophagy activity of epithelial cells, and inhibition of autophagy can significantly reduce IL-13-induced airway mucus hypersecretion, significantly improve airway hyperresponsiveness (AHR), reduce IL-5 levels, reduce eosinophil counts in BALF and attenuate inflammatory pathology in lung tissue (Dickinson et al., 2016a, Dickinson et al., 2016b). In this study, we used immunohistochemistry and Western blotting to detect the autophagy level in the lung tissue of mice. The results showed that quercetin could inhibit the expression of autophagy marker protein in lung tissue of asthmatic mice. At the same time, we carried out *in vitro* experiments to detect the expression of LC3 and Beclin-1 protein in BEAS-2B cells. The experimental results showed that the LC3-II/LC3-I ratio and Beclin-1 in the quercetin treatment group were significantly downregulated, which further verified the inhibitory effect of quercetin on autophagy. These results suggest that the therapeutic effect of quercetin on asthma may be achieved by inhibiting over-activated autophagy. This finding provides theoretical support for further exploring the application of quercetin in the treatment of asthma, and also provides new ideas for the treatment of asthma.

Autophagy induction and suppression are critically governed by the PI3K/Akt/mTOR cascade (Zou et al., 2018). Current scientific literature demonstrates that suppression of PI3K/Akt/mTOR signaling represents an established mechanism for autophagy induction, and autophagy is activated as an intracellular ' scavenger ' mechanism, which is of great significance for maintaining intracellular environmental homeostasis (Yang et al., 2022, Pan et al., 2022). On the contrary, the treatment effect can be achieved by activating this signaling pathway to inhibit the abnormally activated autophagy in the disease (Rogerio et al., 2007, Liu et al., 2021, Cui et al., 2019). This study shows that low-dose quercetin has no significant effect on the PI3K/Akt/mTOR signaling pathway in lung tissue of asthmatic mice. The reason may be that low-dose quercetin is difficult to form a sufficient intensity intervention on this complex signaling pathway. In contrast, high-dose quercetin significantly upregulated p-PI3K, p-Akt and p-mTOR protein levels in lung tissue of asthmatic mice. Based on this phenomenon, we used the PI3K pathway inhibitor LY294002 to verify the regulatory effect of quercetin on the PI3K/Akt/mTOR signaling pathway and autophagy. The results show that the use of LY294002 significantly inhibited the inhibitory effect of quercetin on autophagy, which further supports the involvement of quercetin in the modulation of the PI3K/Akt/mTOR signaling pathway and autophagy. It should be emphasized, however, that the hierarchical causal relationship among PI3K/Akt/mTOR pathway modulation, autophagy normalization, and airway inflammation attenuation remains to be definitively established in this study—an equally tenable alternative interpretation is that quercetin exerts its core anti-inflammatory effects via antioxidant, NF-κB-dependent, or cytokine-modulatory mechanisms, with the detected changes in PI3K/Akt/mTOR signaling and autophagic flux representing downstream sequelae of mitigated inflammation rather than upstream regulatory triggers (Chekalina et al., 2018), thus pointing to the necessity of subsequent investigations to clarify the exact causal interplay of these biological events.

**FIGURE 8:**
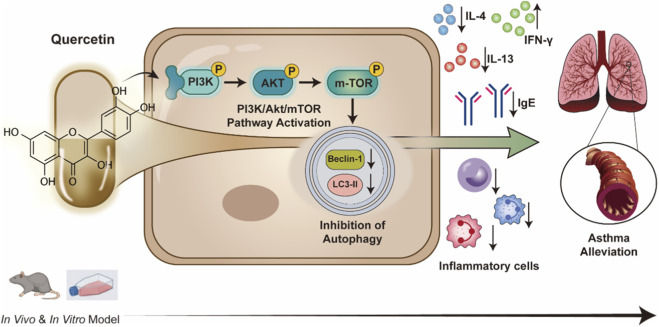
The mechanism by which quercetin regulates the PI3K/Akt/mTOR pathway, autophagy, and airway inflammation in asthmatic mice.

Glucocorticoid is currently the most effective drug for the treatment of asthma. However, chronic treatment could potentially induce multiple adverse reactions, such as osteoporosis and decreased immunity (Gurnell et al., 2021). As a natural flavonoid compound, quercetin has many biological activities such as anti-inflammatory and anti-oxidation, and has relatively few adverse reactions (Aghababaei and Hadidi, 2023). This study demonstrated through *in vivo* and *in vitro* experiments that the therapeutic effect of quercetin on asthma is at least partially achieved by modulating the PI3K/Akt/mTOR signaling pathway, whose regulation contributes to the restoration of excessive autophagy and the attenuation of airway inflammation (Figure 8). These findings provide a new direction and theoretical basis for the treatment of asthma.

However, while this study has achieved certain results, there are inevitably some obvious limitations. First, although the inflammatory model used *in vitro* cell experiments simulates the inflammatory response process during asthma attack to a certain extent, it is still quite different from the complex and changeable physiological and pathological environment in real organisms. Subsequent studies can address this limitation by using co-culture of multiple cell types for verification. Secondly, the OVA-induced asthma model used in this study specifically mimics Th2-high allergic asthma (characterized by Th2 hyperresponsiveness) and cannot represent other asthma endotypes (e.g., Th1-dominant asthma or non-eosinophilic asthma). Additionally, beyond the Th2-high context, quercetin may modulate Th17 cell differentiation and IL-17A secretion via STAT3 signaling, as well as exert inhibitory effects on neutrophilic inflammation in non-eosinophilic asthma subtypes, which warrants validation in more diverse experimental models. Therefore, the conclusions of this paper may not be directly generalized to all clinical asthma scenarios, and additional studies involving clinical samples or more clinically relevant experimental systems are needed to verify their translational potential.

## 5 Conclusion

In conclusion, this study shows that quercetin can effectively reduce airway inflammation and lung tissue damage in OVA-induced Th2-high allergic asthma mice, an effect that is at least partially mediated by the modulation of the PI3K/Akt/mTOR signaling pathway, which contributes to the normalization of excessive autophagy. Low-dose quercetin has a certain therapeutic effect, and the effect of high-dose quercetin is more significant. These results provide new directions and theoretical support for asthma treatment. In the future, through more in-depth research and technical means, it is expected to further clarify the mechanism of action of quercetin, promote its wide application in the clinical treatment of asthma, and bring new treatment options for asthma patients.

## Data Availability

The original contributions presented in the study are included in the article/supplementary material, further inquiries can be directed to the corresponding author.
